# Exploring the Significance of Eosinophil Infiltration in Diagnosis of Psoriasis: A Cross-sectional Analysis 

**DOI:** 10.30699/ijp.2024.2013501.3191

**Published:** 2025-01-10

**Authors:** Maryam Khalili, Afsaneh Kooshesh, Simin Shamsi-Meymandi, Niloofar Mehrolhasani, Rezvan Amiri, Mohammad Rezaei Zadeh Rukerd, Mahin Aflatoonian

**Affiliations:** 1 *Clinical Research Development Unit, Afzalipour Hospital, Kerman University of Medical Sciences, Kerman, Iran*; 2 *Afzalipour Hospital, Kerman University of Medical Sciences, Kerman, Iran*; 3 *Pathology and Stem Cells Research Center, Afzalipour School of Medicine, Kerman University of Medical Sciences, Kerman, Iran*; 4 *Gastroenterology and Hepatology Research Center, Institute of Basic and Clinical Physiology Sciences, Kerman University of Medical Sciences, Kerman, Iran*

**Keywords:** Eosinophil, Pathology, Psoriasis

## Abstract

**Background & Objective:**

There is controversy whether eosinophils are involved in the pathogenesis of psoriasis. This study aims to assess the quantity of eosinophils in pathological specimens obtained from individuals diagnosed with psoriasis.

**Methods:**

cross-sectional and retrospective study 91 skin samples were obtained from patients with diagnosis of psoriasis. Two experienced dermatologists thoroughly reviewed the specimens' demographic characteristics, clinical features, and pathological attributes. Subsequently, eosinophils were counted within all microscopic fields, utilizing a magnification of 200.

**Results:**

Eosinophils were present in approximately 70.3% of the examined samples, with a mean eosinophil count of 2.42±0.63. Although no significant correlation was observed between the clinical subtype and the average eosinophil count, eosinophils were most commonly detected in the cases presenting generalized pustular psoriasis (100%) and vulgaris types (71.11%). Notably, patients exhibiting Munro's microabscess and dilated papillary dermal blood vessels exhibited a significantly higher number of eosinophils (*P*=0.007 and *P*=0.039, respectively). Additionally, a notable association was identified between presence of spongiosis, and eosinophil counts in the pathological samples (*P*=0.04).

**Conclusion:**

Presence of eosinophils may not contradict a diagnosis of psoriasis. Furthermore, a notable association may be observed between the number of eosinophils and presence of spongiosis, dilated dermal papillary vessels, and Munro's microabscess.

## Introduction

Psoriasis is a chronic papulosquamous disease affecting a significant portion of the global population, with an estimated prevalence of 2–3%. It is categorized into various types based on morphology and distribution patterns, including vulgaris, erythrodermic, palmoplantaris, pustular, flexural, and guttate psoriasis (1–3). Among these, vulgaris is the most prevalent, accounting for about 80–90% of all diagnosed cases. Psoriasis can be triggered or exacerbated by multiple factors, such as physical injuries, psychological stress, infections, medications, and smoking (4–5).

Psoriasis vulgaris, the classic form, presents as well-demarcated erythematous plaques with silvery scales, primarily seen on the extensor surfaces and scalp. Pathologically, psoriasis vulgaris is characterized by parakeratosis (retention of nuclei in the stratum corneum), Munro’s microabscesses (collections of neutrophils within the stratum corneum), hypogranulosis (reduced granular layer), Kogoj’s microabscesses (neutrophils in the spinous layer), acanthosis (epidermal thickening), club-shaped elongated rete ridges, suprapapillary thinning (thinning of the epidermis above the dermal papillae), and dilated dermal papillary vessels (6–10).

Pathogenesis of psoriasis involves an overproduction of cytokines associated with specific T-helper cell subsets. Notably, there is an increase in Th1 cytokines, such as interferon-gamma (IFN-γ), interleukin (IL)-2, and tumor necrosis factor-beta (TNF-β), as well as Th22 cytokines (IL-22) and Th17 cytokines (IL-17). Conversely, there is a downregulation of IL-10 (11–13). Antigen-presenting cells, T lymphocytes, and dendritic cells also play a role in psoriasis pathogenesis. Although neutrophils are found in active lesions and contribute to disease activity, they are not considered a primary factor in psoriasis pathogenesis (14–17).

Currently, there is no consensus regarding the role of eosinophils in psoriasis pathogenesis. Some early studies have suggested that finding eosinophils in skin biopsies of psoriatic lesions contradicts the diagnosis, whereas other studies have observed an association between blood eosinophilia and psoriasis, particularly during unstable phases and active disease (18–23). This discrepancy underscores the need for further investigation into the precise role of eosinophils and their potential implications in disease progression.

Given the critical need to distinguish psoriasis from other skin diseases for both effective treatment and assessment of associated comorbidities, this study aimed to evaluate eosinophil counts in skin biopsies of patients with psoriasis. Additionally, it examined potential associations between eosinophil counts and various demographic, clinical, and pathological characteristics of psoriasis. 

## Material and Methods

### Study Design and Population

This retrospective cross-sectional study enrolled 91 patients with psoriasis who underwent skin biopsies. The inclusion criteria included presence of typical clinical presentation of psoriasis, supported by digital clinical information and images of the lesions. Two dermatologists initially reviewed digital data to identify cases meeting the criteria for typical psoriasis, taking into account the patients’ medical histories and clinical images. Demographic and clinical details—such as age, sex, duration of disease, lesion site, and psoriasis subtype—were recorded.

Next, two dermatopathologists reviewed the histopathologic slides , with focus on on particular features , including parakeratosis, acanthosis, spongiosis, Munro’s microabscesses, Kogoj’s microabscesses, suprapapillary thinning, and dilated vessels in the papillary dermis. Spongiosis was classified into three groups: mild (mild intercellular edema with minimal secondary changes), moderate (moderate intercellular edema with significant secondary changes), and severe (severe intercellular edema with blister formation) (24).

Then eosinophils were quantified in all microscopic fields per tissue section at 200× magnification. Only eosinophils with two-lobed nuclei and eosinophilic cytoplasm were counted, and those within blood vessels were excluded. The mean total number of eosinophils, counted separately by the two dermatopathologists, was recorded as the final result. 

Eosinophils were then quantified in all microscopic fields per tissue section at 200× magnification. Only eosinophils with two-lobed nuclei and eosinophilic cytoplasm were counted, excluding those within blood vessels. The mean total number of eosinophils, counted separately by the two dermatopathologists, was recorded as the final result. 

### Statistical Analysis

The data obtained from the study were analyzed using SPSS 16 software (IBM, Armonk, NY, USA). Descriptive statistics, including frequency and percentage, were employed to analyze qualitative data. For quantitative data, the mean and standard deviation were calculated. The Chi-square test or Fisher's exact test was utilized to evaluate the association between the number of eosinophils and the demographic, clinical, and pathological features of the cases.

## Results

The study included a total of 91 psoriasis cases, with 51.64% male and 48.36% female. The mean age of the patients was 40.19 ± 18.30 years, ranging from 5 to 78 years. The average duration of the lesions was 1.78 ± 0.67 years, with a range of 10 days to 15 years. The majority of patients (43.96%) were in the 5th to 6th decades of life, and most of the lesions (96.7%) had a duration of less than five years. Psoriasis vulgaris was the most common type, accounting for 83.6% of the cases, and the lower limbs were the most frequent location for the lesions (45.05%) (Table 1).

The mean number of eosinophil cells observed in the study was 2.42 ± 0.63, ranging from 0 to 11 (Table 2).

The analysis revealed no significant association between the demographic features of the patients, duration and site of the lesions, as well as clinical subtypes of psoriasis, with the eosinophil count (*P*>0.05) (Table 1). Similarly, no significant correlation was found between pathological features such as parakeratosis, acanthosis, suprapapillary thinning, and Kogoj's microabscess, with the eosinophil count (*P*>0.05) (Table 3). However, it is noteworthy that patients with Munro's microabscess and dilated papillary dermis blood vessels demonstrated a significantly higher number of eosinophils (*P*=0.007 and *P*=0.039, respectively). Additionally, there was a significant association between the presence of spongiosis and eosinophil counts in the pathological samples (*P*=0.04) (Table 3).

**Table 1:** Association between demographic and clinical features of the patients with psoriasis with eosinophilic count

Variables		Frequency (%)	Eosinophil count (mean ± SD)	P-value
Sex	Male	47 (51.64)	2.96±0.48	0.05
	Female	44 (48.36)	1.72±0.36	
Age groups (years)	< 20	16 (17.58)	1.27±0.42	0.12
	20-40	23 (25.27)	3.23±0.67	
	41-60	40 (43.96)	2.0±0.45	
	> 60	12 (13.19)	3.25±1.08	
Duration of disease (years)	<5	88 (96.70)	2.08±0.25	0.13
	5-10	2 (2.2)	0.0± 0.0	
	11-15	1 (1.1)	4	
Site of lesions	Head and neck	4 (4.4)	1.5±0.86	0.96
	Trunk	26 (28.57)	2.46±0.83	
	Upper limb	19 (20.88)	2.35±0.60	
	Lower limb	41 (45.05)	2.48±0.47	
	Genitalia	1 (1.1)	4	
Clinical subtypes	Vulgaris	76 (83.6)	2.22±0.37	0.07
	Palmoplantaris	6 (6.6)	1.83±0.64	
	Generalized pustular	3 (3.3)	5.40±1.32	
	Flexural	3 (3.3)	3	
	Guttate	5 (5.5)	1.33±0.66	

**Table 2:** Frequency of eosinophils in skin biopsy of patients with psoriasis

Eosinophil count	Frequency	Percentage
0	27	7.29
1-2	32	12.34
3-5	22	24.2
> 5	10	11

**Table 3:** Association between pathological features of the patients with psoriasis with eosinophilic count

Variables		Frequency (%)	Eosinophil count (mean ± SD)	P-value
Parakeratosis	Focal	47 (51.6)	2.65 ± 0.43	0.515
	Confluent	40 (44)	2.27 ± 0.36	
	Absent	2 (4.4)	0.0± 0.57	
Acanthosis	Present	86 (94.5)	2.37 ± 0.27	0.407
	Absent	5 (5.5)	3.50 ± 2.21	
Suprapapillary thinning	Present	81 (89)	2.42 ± 0.28	0.983
	Absent	10 (11)	2.44 ± 1.14	
Munro's microabscess	Present	47 (51.6)	3.12 ± 0.41	0.007
	Absent	44 (48.4)	1.64 ± 0.33	
Spongiosis	Mild	43 (47.3)	2.32 ± 0.39	0.043
	Moderate	21 (23.1)	3.33 ± 0.71	
	Severe	5 (5.5)	4.0 ± 0.37	
	Absent	22 (24.2)	1.3 ± 0.30	
Dilated papillary dermis vessels	Present	51 (56)	2.92 ± 0.38	0.039
	Absent	40 (44)	1.76 ± 0.37	
Kogoj's microabscess	Present	14 (15.4)	3.21 ± 0.83	0.302
	Absent	77 (84.6)	2.28 ± 0.29	

## Discussion

Eosinophils, characterized by their eosinophilic granules and bilobed nucleus, are pro-inflammatory cells. Their role in the pathogenesis of psoriasis is thought to involve the release of inflammatory mediators, such as eosinophilic cationic protein (ECP) and major basic protein (MBP), which can activate lymphocytes. Studies have shown an increase in chemotactic factors, such as platelet-activating factor (PAF), complement receptor (C5a), and leukotriene B4, in the epidermis of psoriatic lesions. These factors can prompt eosinophil migration, suggesting their possible role in the pathophysiology of the disease (21).

In the present study, eosinophils were identified in 70.3% of the examined skin biopsies, with a mean count of 2.42±0.63. These findings contrast with the study by Rosa* et al.*, which reported eosinophils in only 18% of psoriatic cases, with a mean eosinophil count of 0.3 (25). In another study by Penn* et al.*, 46% of skin biopsies from psoriatic patients exhibited eosinophils, with a mean count of 1.04; most cases had one or two eosinophils (34%) (26). Interestingly, our current study demonstrated a similar pattern, with the majority of cases (35.2%) having 1–2 eosinophils. Additionally, our findings align with Penn* et al.*, as no significant correlation was observed between eosinophil count and the biopsy site (26).

Our study results showed a significant positive correlation between the presence of Munro’s microabscess and the dilatation of dermal papillary vessels with the mean eosinophil count (3.12 ± 0.41 and 2.92 ± 0.38, respectively). Furthermore, a significant positive association was found between spongiosis severity and eosinophil count. Notably, cases without spongiosis had the lowest mean eosinophil count (1.3±0.30). Penn* et al.* also reported the presence of eosinophils exclusively in spongiotic cases but found no significant relationship between spongiosis severity and eosinophil count. Our findings differ from that study, as we observed a significant positive link between spongiosis severity and eosinophil count (26).

In our current study, although no significant association was identified between the clinical subtype of psoriasis and the mean eosinophil count, eosinophils were most frequently detected in generalized pustular psoriasis (GPP) and psoriasis vulgaris (100% and 71.11% of cases, respectively). Moreover, the highest mean eosinophil count was observed in GPP (5.40 ± 1.32), whereas the lowest mean eosinophil count was seen in guttate psoriasis (1.33±0.66). These findings are consistent with a previous study by Rosa* et al.*, which also revealed the highest eosinophil counts in GPP (5.40±1.32) and psoriasis vulgaris (2.22±0.37) (25). Two studies by Rosa and Chau* et al.* reported the greatest eosinophil counts in vulgaris-type psoriasis (15.45%), while the lowest counts were observed in palmoplantaris, erythrodermic, and guttate forms, with respective values of 0%, 1.13%, and 1.13% (25,27). Three other studies investigating pustular palmoplantar psoriasis demonstrated varied eosinophil percentages ranging from 11.8% to 86.4% (28–30). In our research, 66.6% of palmoplantar psoriasis patients had eosinophils. Kardaun* et al.* found a higher percentage of eosinophils in GPP compared to acute generalized exanthematous pustulosis (AGEP), reporting 7.1% eosinophils in AGEP (31). In contrast, in our study, eosinophils were present in all GPP cases. These GPP cases had a history of psoriasis with lesion exacerbation following abrupt cessation of systemic psoriasis treatments and no history of other medication use prior to disease onset. Mansur* et al.* also reported peripheral blood eosinophilia in 41% of GPP cases, suggesting that the mere presence or absence of eosinophils may not suffice to distinguish AGEP from GPP. Other factors, such as a history of psoriasis, sudden discontinuation of psoriatic medications, and any pharmacological history preceding the onset of pustular lesions, may play a more critical role. However, the small number of GPP cases in our study underscores the need for further large-scale studies.

In another study by Lundin* et al.*, patients with generalized psoriasis, especially those with arthritis, showed peripheral blood eosinophilia. They also noted elevated ECP and MBP in the peripheral blood and skin biopsies of psoriatic patients, whereas these markers were not increased in healthy controls. Additionally, they reported higher ECP levels in unstable disease, marked by new lesions or rapid disease progression, while slowly progressive cases exhibited lower ECP levels. These findings indicate that disease activity (stable or unstable) and psoriasis type can affect eosinophil presence and percentage in peripheral blood and skin lesions (21).

The principal limitation of this study is the absence of positive (e.g., allergic contact dermatitis) and negative (e.g., lichen planus) control groups. Consequently, future research should involve case-control studies with larger sample sizes to validate our findings.

## Conclusion

In our study, the majority of psoriasis cases exhibited the presence of eosinophils. Furthermore, we observed a significant positive correlation between the eosinophil count and the severity of spongiosis, as well as the presence of dilated dermal papillary vessels and microabscess of Munro in the skin biopsies of psoriatic lesions. These findings suggest a potential association between eosinophils and the pathological features of psoriasis, indicating their involvement in the disease process.
